# Optimizing Autologous Serum Tear Therapy for Dry Eye Disease: Strategies and Innovations

**DOI:** 10.3390/jcm15031181

**Published:** 2026-02-03

**Authors:** Konstantinos Christodoulou, Brayden Buras, Sotiria Palioura

**Affiliations:** 1 Department of Ophthalmology, Bascom Palmer Eye Institute, University of Miami Miller School of Medicine, Miami, FL 33136, USA; konstantinosxristodulu@gmail.com; 2 Beauty of Sight Eye Bank, Bascom Palmer Eye Institute, Miami, FL 33136, USA; braydenjburas@gmail.com; 3 University of Mississippi, Oxford, MS 38677, USA

**Keywords:** dry eye disease, autologous serum tears, concentration, diluents, clotting, centrifugation, storage

## Abstract

Autologous serum (AS) tears are an effective therapeutic option for advanced DED, mimicking the biochemical composition of natural tears. However, the absence of universally accepted guidelines has resulted in variability in AS tear concentration, diluents, processing of collected blood, and storage conditions, raising questions regarding the optimal parameters for AS tear use. This perspective provides a framework to inform clinical implementation and to guide future research on AS tear therapy optimization. PubMed, Scopus, and the Cochrane Library were searched for English-language articles from January 2022 through September 2025 using the terms “autologous serum,” “dry eye disease,” “dry eye syndrome,” “dry eye,” and “DED.” Evidence suggests that AS tears diluted to 20% are widely used for moderate DED, whereas higher concentrations may provide faster, more pronounced and more durable improvements, particularly in severe cases. Levofloxacin-containing eye drops, artificial tears without emphasis on a specific component, sodium hyaluronate (SH)-containing eye drops, cyclosporine A (CsA)-containing ultra-nano emulsions, and methylcellulose have been investigated as alternatives to conventional diluents. Standardization of clotting, centrifugation and storage parameters is expected to enhance efficacy of AS tears and ensure stability of growth factors. Combination with estrogen replacement therapy in perimenopausal women or with topical insulin eye drops, as well as perioperative prophylactic use in patients with graft-versus-host disease (GVHD)-associated dry eye undergoing cataract surgery, represent emerging applications of AS tears that demonstrate potential to improve therapeutic outcomes. Overall, this perspective highlights the need for consensus protocols, supports severity-based concentration tailoring, and notes that diluents and processing methods require further refinement.

## 1. Introduction

Dry eye disease (DED) is defined as “a multifactorial, symptomatic disease characterized by a loss of homeostasis of the tear film and/or ocular surface, in which tear film instability and hyperosmolarity, ocular surface inflammation and damage, and neurosensory abnormalities are etiological factors” [1]. The prevalence of dry eye disease, stratified by age and sex, ranges from 5.4% to 44.2% using the Tear Film & Ocular Surface Society’s Dry Eye Workshop (TFOS DEWS) II criteria, and from 4.7% to 62.9% based on symptoms and signs [2]. Mechanistically, DED comprises two principal subtypes, aqueous deficient and evaporative dry eye, each associated with disorders of the lacrimal and meibomian glands, respectively [3]. Clinical manifestations range from mild irritation and conjunctival hyperemia to severe ocular pain, fluctuating vision, conjunctival scarring, and corneal complications [3,4,5]. Advanced disease might impair vision, reduce productivity, and pose substantial therapeutic challenges [3,4,6].

Emerging evidence supports the therapeutic potential of autologous serum (AS) tears in advanced DED [7,8]. AS tears are prepared by drawing the patient’s blood and separating the serum from the cellular components. Their biochemical composition resembles that of natural tears, promoting ocular surface lubrication, epithelial healing, and corneal nerve regeneration, while also exerting anti-inflammatory, anti-apoptotic, and anti-microbial effects [9,10,11].

Despite increasing clinical use, universal guidelines for AS tears have not been established [12]. The TFOS DEWS III Management and Therapy Report underscores the importance of developing comprehensive standards to optimize AS tear therapy [13]. The therapeutic efficacy of AS tears depends on dilution ratio, diluent type, and processing of collected blood, including clotting and centrifugation parameters; however, substantial heterogeneity persists across studies and routine practice [14,15]. Storage conditions also influence the biochemical composition and clinical effectiveness of AS tears, but data on storage duration and temperature remain incomplete and variable [15,16]. This perspective integrates recent insights addressing these determinants, and provides evidence-based guidance to support the development of a consensus protocol and to optimize AS tear therapy for patients with DED.

## 2. Materials and Methods

This perspective review aims to synthesize and critically assess the most recent literature on optimizing AS tears in patients with DED.

For this structured perspective, a literature search was conducted using PubMed, Scopus, and the Cochrane Library to identify articles published in English between January 2022 and September 2025. Our search strategy used the following keywords: “autologous serum,” “dry eye disease,” “dry eye syndrome,” “dry eye,” and “DED.” Inclusion criteria comprised original articles, randomized and nonrandomized controlled trials, comparative studies, observational studies, and systematic and comprehensive reviews focusing on AS tear therapy in DED. In addition to database search, the reference lists of relevant articles were manually screened to identify further studies.

For the purposes of this perspective, the analysis is focused exclusively on findings related to DED and is organized into four sections: concentration, diluents, processing of collected blood and storage conditions, and emerging applications. Each section examines recent evidence and includes directions for future research or clinical implementation.

As previously mentioned, there are no universal regulatory guidelines for AS tear therapy, and considerable methodological variability exists across published studies, encompassing differences in concentration, diluents, processing of collected blood, and storage conditions. This perspective study adopts a structured approach by examining each of these parameters independently. While acknowledging the potential interplay among them, this strategy allows for focused evaluation of these parameters, consistent with prevailing approaches, thereby contributing to progress toward establishing consensus on the optimal AS tears utilization.

## 3. Concentration

AS tears are prepared at concentrations ranging from 20% to 100% for the treatment of DED [10]. Evidence that transforming growth factor β (TGF-β) is approximately five-fold more concentrated in serum than in natural tears led to the adoption of a 20% dilution to mitigate potential anti-proliferative effects of TGF-β, thereby establishing 20% AS tears as the most frequently employed concentration in both clinical studies and routine practice [16]. Lower concentrations are safe and promote epithelial cell proliferation in patients with DED; however, dilution aimed at limiting TGF-β also reduces factors with potential regenerative effects [17,18,19]. Higher concentrations are well-tolerated and enhance epithelial migration and extracellular matrix deposition, potentially fostering a more favorable environment for ocular surface repair [17,19]. In moderate DED, the efficacy of 20% AS tears appears comparable to that of 50% AS tears, whereas 50% AS tears are often preferred for severe disease [6,20]. Nevertheless, there is currently no consensus regarding the optimal concentration of AS tears for achieving maximal therapeutic efficacy in DED [18].

Over the past 3 years, 16 clinical studies have examined AS tears at varying concentrations as a therapeutic option for DED, reflecting evolving trends in both research and clinical practice [6,18,21,22,23,24,25,26,27,28,29,30,31,32,33,34,35]. Of these, 10 studies employed 20% AS tears [22,23,26,28,29,30,32,33,34,35], four used higher concentrations [6,18,24,25,27], one included both 20% and 50% concentrations [21], and one directly compared 20% with 50% concentration [31].

It is important to note that herein outcomes from relevant studies [6,18,21,22,23,24,25,26,27,28,29,32,33,34,35] are reported relative to baseline, as presented in the original publications. Study [31] is reported relative to the comparator arm, in accordance with its original design. Additionally, Neubauer et al. evaluated AS tears diluted to 20% with 0.9% saline solution in patients previously treated with AS tears diluted to 20% with methylcellulose; however, results for subjective symptoms, Schirmer’s test, tear break-up time (TBUT), ocular surface staining (OSS), and tear meniscus height (TMH) were reported only in the context of the comparison between diluents and therefore the effects of 20% AS tears cannot be interpreted relative to pretreatment [30] (Table 1).

### 3.1. 20% Autologous Serum Tears

Of the 10 studies that employed 20% AS tears, seven assessed changes in subjective symptoms, six of which reported improvement [23,26,29,32,33,34], whereas one did not show a benefit [28].

Tear production was evaluated in nine studies using Schirmer’s test. Of the seven studies that conducted the test without anesthesia, five reported increased tear production [23,26,29,32,34], one showed a non-significant increase [33], and one showed no improvement [28]. Two studies did not specify whether anesthesia was used and both reported improvements [22,35].

TBUT was examined in nine studies, with eight reporting an increase following treatment [22,23,26,28,29,32,34,35], whereas one showed an increase that was not statistically significant [33].

OSS was assessed in eight studies using varying methods. Five studies used corneal fluorescein staining alone [22,23,26,29,35], two corneal fluorescein and conjunctival lissamine green staining [28,34], and one employed corneal and conjunctival staining without specifying the dyes [33]. All studies reported improvement, although the study using unspecified dyes showed a non-significant reduction.

Conjunctival epithelial parameters were evaluated in two studies using conjunctival impression cytology (CIC). Kang et al. assessed CIC metaplasia grade and goblet cell density, and found no significant change [28], while Zheng et al. evaluated epithelial morphology and inflammation, and observed an improvement [29].

Corneal sensation, measured by blink reflex, was evaluated in one study, which observed improvement following treatment [23].

Visual acuity (VA) was compared with baseline in four studies. One reported improved visual acuity following treatment [35], whereas the other three found no statistically significant change, although two of these three mentioned a slight improvement [26,28,33].

Inflammatory and biochemical parameters of patients’ tears were evaluated in two studies. Following treatment, Wang et al. reported reductions in tear inflammatory factors, including tumor necrosis factor alpha (TNF-α), interleukin 1 beta (IL-1β), and interleukin-6 (IL-6) [23]. Li et al. observed decreased expression of TNF-α, IL-1β, prostaglandin E2 (PGE2), and vascular endothelial growth factor (VEGF) and increased expression of tear components, namely lysozyme, lactoferrin, albumin, and secreted immunoglobulin alpha (sIgA) [22].

Adverse events and complications were examined in six studies. Three studies documented no adverse effects [28,33,34], while the remaining three reported only infrequent, mild events, including ocular irritation and itching, conjunctival hyperemia, photophobia, and headache, none of which were severe [23,29,35].

### 3.2. Higher-Concentration Autologous Serum Tears

Of the four studies employing AS tears at concentrations above 20%, two investigated 50% AS tears [6,27]. Schirmer’s test with anesthesia was assessed in one study, which reported improvement [6]. TBUT increased in both studies [6,27]. OSS improved in both studies, with Bachtalia et al. evaluating corneal fluorescein and conjunctival lissamine green staining, whereas Shaheen et al. assessed corneal fluorescein staining only [6,27]. Confocal microscopy parameters of corneal nerve health were evaluated in one study, which demonstrated improvement [6]. VA was higher than baseline in both studies [6,27]. Bachtalia et al. reported no clinical adverse events or infections, although microbiological analysis of the used vials identified Gram-negative bacteria [6,27].

The remaining two studies examined 100% AS tears [18,24,25]. Subjective symptoms of dry eye were reduced in both studies. Schirmer’s test without anesthesia and TBUT improved in both studies [18,24], though not significantly in one study [24]. Corneal and conjunctival staining were assessed in both studies using the Oxford grading system and showed a decrease compared to baseline [18,24]. Conjunctival hyperemia was evaluated in one study and demonstrated a decrease following treatment [18]. Meibomian gland parameters, including quality and expressibility, were measured in both studies, with no statistically significant changes observed [18,24]. VA was evaluated in one study and demonstrated improvement [18]. Both articles assessed growth factor levels in AS tears but reported outcomes only in comparison with another treatment arm, limiting interpretation of the effects of AS tears alone [18,25]. Adverse events were examined in both studies. One study reported no adverse effects [18], whereas the other documented only rare, mild events, including ocular irritation and sticky sensation [24].

### 3.3. 20% and 50% Autologous Serum Tears

One study evaluated AS tears at concentrations of 20% and 50% together with sodium hyaluronate (SH)-containing eye drops in dry eye following phacoemulsification cataract surgery, with SH-containing eye drops alone serving as the control [21]. Compared to the control group, improvements were observed in subjective symptoms, Schirmer’s test without anesthesia, TBUT, corneal and conjunctival staining, conjunctival hyperemia, and CIC scores. However, the study did not provide comparative data to clarify whether different AS concentrations confer distinct clinical benefits [21].

Another study directly compared 20% with 50% AS tears in patients with moderate and severe DED [31]. Among patients with moderate DED, both concentrations produced statistically significant improvements in subjective symptoms, Schirmer’s test with anesthesia, TBUT, and OSS. Improvements in Schirmer’s test occurred more rapidly in the 50% group, and by week 12, the between-group difference reached statistical significance. At the same time point, OSS was also significantly reduced in the 50% group compared with the 20% group. In patients with severe DED, subjective symptoms improved significantly in both groups, but more rapidly in the 50% group. Schirmer’s test with anesthesia, TBUT, and OSS improved significantly only in the 50% group. No adverse events were reported. After the end of the treatment, worsening of symptoms was more frequent among patients previously treated with 20% AS tears in both moderate and severe DED [31].

In addition, in a comprehensive review of patients with moderate-to-severe DED, Bachtalia et al. found substantial overlap in outcomes between 20% and 50% AS tears in subjective symptom relief [36]. It was further noted that a single study evaluating 50% AS tears demonstrated marked improvements in ST, TBUT and OSS, in contrast to the more modest gains in multiple studies of 20% AS tears [6]. VA improved slightly in the 50% AS tears study but remained unchanged in studies of 20% AS tears [36].

### 3.4. Optimal Concentration of Autologous Serum Tears

Collectively, studies published over the past three years indicate that 20% is the most extensively investigated concentration of AS tears, consistently improving both subjective symptoms and objective measures in DED. Research on higher-concentration AS tears and comparative evaluations of 50% with 20% formulations remain limited but suggest faster, more pronounced, and more durable improvements. Accordingly, our findings support the rationale for the use of 20% AS tears in moderate DED and higher concentrations in advanced DED, consistent with current practice.

The lack of a consensus standardized protocol for the processing of collected blood, including clotting and centrifugation parameters, together with variability in diluents, limits definitive conclusions between 20% and higher-concentration AS tears across existing studies. Additional studies directly comparing different concentrations of AS tears are required, employing consistent processing of collected blood and uniform diluents, and systematically assessing the levels of growth factors in relation to clinical outcomes, to determine the optimal concentration for varying severities of DED.

## 4. Diluents

AS may be diluted with preservative-free normal saline, balanced salt solution (BSS), or other sterile, preservative-free solutions that are compatible with ocular use [12]. Saline solutions were the first diluents to be described and remain the most commonly used [30]. BSS became a well-established option after Liu et al. proposed a refined protocol for the production of AS tears, demonstrating benefits on epithelial cell proliferation [14,17].

Recent studies have explored alternative diluents with the aim of optimizing the therapeutic efficacy of AS tears in DED. Over the past 3 years, formulations have included levofloxacin-containing eye drops [22,23], artificial tears without emphasis on any specific component [6], 0.1% SH-containing preservative-free eye drops [21,28], 0.05% cyclosporine A (CsA)-containing ultra-nano emulsions [37], and 0.5% methylcellulose [30] (Table 2).

### 4.1. Levofloxacin-Containing Eye Drops

Li et al. reported improved Schirmer’s test scores, TBUT, and OSS in perimenopausal women with severe DED following treatment with AS tears diluted to 20% with levofloxacin-containing eye drops. Ocular surface inflammatory factors, including TNF-a, IL-1β, VEGF, and PGE2 were decreased, whereas tear components, namely lysozyme, lactoferrin, albumin, and sIgA were increased [22].

Wang et al. investigated the effects of diluting AS tears with levofloxacin-containing eye drops, alongside supplemental artificial tear instillation in patients with dry eye following cataract surgery [23]. Subjective symptoms, Schirmer’s test without anesthesia, TBUT, and corneal staining were improved compared with baseline and artificial tear monotherapy. Tear inflammatory factors, including TNF-α, IL-1β, and IL-6 were reduced, while adverse effects were mild and infrequent. Levofloxacin-containing eye drops were proposed to reduce cyclooxygenase activity and inflammatory mediator secretion, exert bactericidal effects via inhibition of bacterial topoisomerase IV and gyrase, and thereby mitigate inflammation, supporting their potential role as AS tears diluents in post-cataract dry eye [23].

Evidence suggests that diluting AS tears with levofloxacin-containing eye drops reduces inflammation and enhances clinical outcomes, and may confer particular benefit in perimenopausal women and patients following cataract surgery. Clinical studies should compare AS tears diluted with levofloxacin-containing eye drops to those diluted with saline solutions or BSS, with possible emphasis on perimenopausal women and postoperative patients to further substantiate these findings. In addition, future longer-term studies and clinical implementation should assess the potential risk of antimicrobial resistance and ocular surface dysbiosis associated with prolonged topical antibiotic exposure, an issue that was beyond the scope of the included studies [38].

### 4.2. Artificial Tears Without Emphasis on a Specific Component

Bachtalia et al. demonstrated that AS tears diluted to 50% with artificial tears improved Schirmer’s test with anesthesia, TBUT, corneal and conjunctival staining, and in vivo confocal microscopy (IVCM) parameters of corneal nerve health compared with baseline and with artificial tears monotherapy in patients with severe DED, and reported no adverse effects despite several instances of Gram-negative contamination of used vials [6].

A systematic review and meta-analysis of AS tears diluted with artificial tears provided additional evidence supporting the therapeutic effect of this formulation, demonstrating significant improvements after treatment compared with artificial tears alone, including enhanced symptom relief, Schirmer’s test scores, TBUT, and OSS [19].

### 4.3. Sodium Hyaluronate-Containing Eye Drops

SH-containing eye drops represent an important therapeutic option for DED, offering good tolerability and efficacy [13]. SH enhances viscosity and water retention, exerts anti-inflammatory activity, and stabilizes the ocular surface and tear film through inactivation of the CD44 adhesion molecule [39]. Physiologically, SH can adopt a large, flexible conformation that permits the free diffusion of small molecules while delaying the diffusion of larger ones, and, collectively with its therapeutic effects, SH is believed to supplement the retention and efficacy of the epitheliotropic factors in AS tears, thereby improving treatment outcomes and reducing the frequency of instillations [30,40].

Kang et al. evaluated the effects of AS tears diluted to 20% with 0.1% SH-containing preservative-free eye drops in patients with primary Sjögren’s syndrome-associated DED [28]. Compared with baseline, TBUT, corneal staining, and conjunctival staining improved. In contrast, subjective symptoms, Schirmer’s test without anesthesia, VA, and CIC metaplasia grade and goblet cell density showed no significant change [28].

Jiang et al. investigated the effects of AS tears diluted to 20% or 50%, together with separate instillations of 0.1% SH-containing eye drops, in patients with DED after phacoemulsification cataract surgery. It is important to note that, although SH was not used as a diluent, combining AS tears with 0.1% SH-containing eye drops resulted in greater symptom relief, improved Schirmer’s test without anesthesia, TBUT, corneal and conjunctival staining, conjunctival hyperemia, and CIC scores, compared with both baseline and SH-containing eye drops alone [21].

In vitro, Sharma et al. evaluated the effects of AS tears diluted with 0.1% SH-containing eye drops in human primary limbal epithelial cell cultures treated with IL1-β to induce proinflammatory activity [37]. The combination exhibited anti-inflammatory effects and enhanced cell viability, providing results comparable to SH alone, while maintaining stable growth factor levels. Additionally, AS tears diluted with 0.1% SH had a pH similar to that of the natural tear film, which may contribute to improved ocular comfort, while density and osmolality remained within the tolerable limits for ocular use [37].

Recent evidence indicates that dilution with SH may enhance the therapeutic efficacy of AS tears in DED without compromising safety. However, further studies are needed to draw definitive conclusions. Future studies should directly compare AS tears diluted with SH to AS tears diluted with conventional media, such as saline solutions and BSS, to determine whether this combination represents a synergistic formulation with superior therapeutic potential.

### 4.4. Cyclosporine A-Containing Ultra-Nano Emulsions

CsA is an effective treatment for moderate-to-severe DED, providing symptom relief, increasing tear production, and reducing ocular surface inflammation and staining without the adverse effects associated with corticosteroids. These clinical benefits are mediated by the anti-inflammatory and immunomodulatory effects of CsA, which include inhibition of T-cell activation and recruitment, and further reduction in proinflammatory cytokine production [37].

In the same study evaluating AS tears diluted with 0.1% SH-containing eye drops, Sharma et al. examined the in vitro effects of AS tears diluted with 0.05% CsA-containing ultra-nano emulsions in IL-1β-treated human primary limbal epithelial cell cultures [37]. This combination reduced proinflammatory markers and improved cell viability to a degree comparable to CsA alone, while preserving stable growth factor levels and a tear film-like pH, with density and osmolarity within the tolerable range for ocular application. The use of ultra-nano emulsions provided enhanced stability, bioavailability and non-irritating properties, facilitating integration of AS with the aqueous phase of the emulsion without disrupting the cyclosporine-containing oil phase. Consequently, Sharma et al. suggested that dilution with CsA-containing ultra-nano emulsions may enhance the therapeutic efficacy of AS tears, thereby improving ocular surface recovery, alleviating symptoms of DED, reducing the number of instillations and providing sustained long-term benefit [37].

Despite promising in vitro findings, no clinical study over the past 3 years has evaluated AS tears diluted with CsA in patients with DED. Further clinical evaluation is warranted to assess efficacy, safety, and long-term potential.

### 4.5. Methylcellulose

Neubauer et al. evaluated patients with severe DED who were treated with AS tears diluted to 20% with methylcellulose for 6 months, followed immediately by a 6-month course of AS tears diluted to 20% with 0.9% saline solution [30]. Despite the change in diluent, subjective symptoms remained improved compared with pretreatment. The authors suggested that the 0.9% saline solution represents a more cost-effective diluent, although it requires a higher dosage [30].

## 5. Processing of Collected Blood and Storage Conditions

Despite their central role in defining the biochemical properties of AS tears, processing of collected blood and storage conditions have received limited attention over the past 3 years, as research has largely focused on formulation-related innovations.

### 5.1. Processing of Collected Blood

Multiple protocols exist for the clotting and centrifugation steps in the preparation of AS tears, with the method proposed by Liu et al. being the most widely employed [11,17]. Liu et al. demonstrated that prolonged clotting at room temperature increases the concentration of beneficial growth factors, and higher centrifugal forces reduce TGF-β1 while increasing epidermal growth factor (EGF) and vitamin A. Based on these findings, they recommended clotting for 120 min at room temperature followed by centrifugation at 3000× *g* for 15 min [14].

Over the past 3 years, 13 clinical studies evaluating AS tears in DED have reported detailed blood-processing protocols [6,18,21,22,23,24,26,28,29,30,31,32,33]. Clotting conditions have been relatively consistent, with most protocols allowing clotting at room temperature for 2 h [6,21,24,26,28]. Two studies used shorter clotting periods of 45 min or 1 h [18,31], one used 31 °C for 2 h [33], and one omitted clotting prior to centrifugation [30]. In contrast, centrifugation parameters remain more heterogeneous. Centrifugation was most commonly performed at 3000× *g* for 15 min [6,24,26], although some studies applied a centrifugal force of 3500× *g* for 15 [28] or 3000× *g* for 30 min [33]. When reported in revolutions per minute (rpm), 3000 rpm for 15 min was the most common setting [22,23,29], with others ranging from 2500 to 4000 rpm for 10 min [18,21,30,31,32]. Centrifugation temperature was specified in three studies and ranged from 4 °C to 20 °C and room temperature [24,30,33]. Notably, one study reported performing centrifugation twice, first for venous blood and then for serum alone [18].

The presence of multiple protocols and the lack of studies directly comparing alternative blood-processing methods indicate that, over the past 3 years, research aimed at providing optimal guidelines for clotting and centrifugation parameters has been limited. Such guidelines are expected to enhance the efficacy of AS tears and improve cross-trial comparability for other efficacy-determining factors. In addition, the inconsistent application of the method proposed by Liu et al., although providing an evidence-based framework for maximizing beneficial growth factors while reducing TGF-β1, suggests that more effective options may exist. Further research is needed to determine whether strict adherence to this protocol translates into superior therapeutic outcomes.

### 5.2. Storage Conditions

The effects of storage conditions on the biochemical composition of AS tears have been studied frequently [16]. The study by Tsubota et al., which established the clinical use of AS tears, assessed the impact of storage duration and temperature on factors such as TGF-β, a regulator of ocular homeostasis with potential anti-proliferative effects at high concentrations, and EGF, which promotes epithelial migration and proliferation while limiting apoptosis [16,41]. Subsequent studies, including the aforementioned, landmark study by Liu et al., expanded this analysis to additional beneficial factors, such as platelet-derived growth factor A/B (PDGF-AB) [14]. However, recent evidence indicates that comprehensive data covering all clinically relevant storage conditions remain incomplete [16].

Among recent publications evaluating autologous serum tears, 10 studies reported details regarding storage conditions [18,21,24,26,27,28,29,30,33,35]. In the majority, patients were instructed to maintain vials in active use at 4 °C for short-term storage, while unopened containers were preserved at −20 °C for longer-term use [18,21,24,26,28,30,33,35]. Two studies reported alternative practices, including long-term storage at −30 °C or −80 °C [27,29].

Furthermore, Metheetrairut et al. assessed the effect of storage temperature and duration on the biochemical composition of 20% AS tears [33]. At 4 °C, TGF-β1, EGF, and PDGF-AB remained stable after 1 week, whereas fibronectin increased modestly. Storage at −20 °C was associated with modest increases in TGF-β1 and EGF after 1 month and in PDGF-AB after 3 months, while fibronectin remained stable [33].

Jirsova et al. measured TGF-β1, EGF, and insulin-like growth factor 1 (IGF-1), in 20% and 100% AS tears stored at 4–8 °C for up to 8 days and at −20 °C, −80 °C, or −156 °C for up to 7 months [16]. TGF-β1 levels remained stable across storage conditions, except in 100% AS tears at 4–8 °C on day 8, where a decrease was observed. EGF remained highly stable under all conditions, with a small, non-significant increase over time at lower temperatures. IGF-1 demonstrated reduced stability under various conditions. When using 20% AS tears, significant decreases were observed after day 8 at 4–8 °C, on month 4 at −20 °C, and after 7 months at −156 °C. When using 100% AS tears reductions occurred after day 7 at 4–8 °C, after 4 months at −156 °C, and after 7 months at all temperatures. Based on these findings, the authors endorsed the current practice of storing 20% AS tears up to 7 days at 4–8 °C, proposed that 20% AS tears can be stored for as long as 7 months at −80 °C without compromising stability, and presented data that may inform the storage of higher-concentration AS tear formulations in clinical practice [16].

Current evidence indicates that short-term storage at 4–8 °C for up to 7 days, provides a solid foundation for practical guidelines. In contrast, long-term storage practices are more heterogeneous, and emerging studies suggest novel strategies that may allow extended preservation of AS tears without compromising stability. Future research should aim to define optimal long-term storage conditions, which, combined with the established short-term protocols, will form comprehensive guidelines for the storage of AS tears.

## 6. Emerging Applications

### 6.1. Combination of Autologous Serum Tears with Estrogen Replacement Therapy for Dry Eye Disease in Perimenopausal Women

The structure and function of the meibomian gland tissues and cells, which secrete lipid components essential for maintaining ocular surface health, are partly regulated by estrogen. Consequently, hormonal changes during perimenopause are associated with an increased incidence of DED. However, standard ophthalmic therapy alone is often insufficient to address the ocular symptoms in this population [22].

Li et al. reported that combination therapy with AS tears and estrogen replacement therapy (ERT) resulted in significantly greater improvements in Schirmer’s test scores, TBUT, and OSS than either treatment alone or standard ophthalmic therapy in perimenopausal women with severe DED. Ocular surface inflammatory factors were significantly reduced, whereas lysozyme, lactoferrin, albumin, and sIgA increased [22].

Findings from Li et al. suggest that combination of AS tears and ERT may offer a valuable therapeutic approach for this specific population. Further studies are needed to confirm this synergistic effect and to define evidence-based treatment protocols.

### 6.2. Combination of Autologous Serum Tears with Off-Label Ophthalmic Application of Insulin Eye Drops for Dry Eye Disease

Burgos-Blasco et al. conducted a retrospective case series evaluating the effects of off-label topical insulin eye drops as an adjunctive therapy in 16 patients with DED, 12 of whom were concurrently using AS tears among other treatments [42]. The investigators observed improvements in subjective symptoms, corneal staining, and conjunctival hyperemia and reported that insulin eye drops promote regeneration of corneal epithelium, highlighting the need for prospective studies to confirm these preliminary findings. However, no definitive conclusions can be drawn from this study regarding a potential synergistic effect of AS tears and topical insulin eye drops; standard therapies were continued for ethical reasons, and patients were receiving a variety of formulations rather than AS tears alone [42].

### 6.3. Autologous Serum Tears as Prophylaxis Before and After Cataract Surgery in Patients with Graft-Versus-Host Disease-Associated Dry Eye

Roca et al. retrospectively assessed cataract surgery outcomes in patients with graft-versus-host disease (GVHD)-associated dry eye, who received perioperative treatment, with pooled human immune globulin eye drops, 50% AS tears, or 1% preservative-free methylprednisolone eye drops, alone or in combination with each other [43]. The study reported a reduction in ocular surface complications compared with rates previously described in literature for patients with GVHD undergoing cataract surgery. Although these findings suggest that this method of utilizing AS tears may represent a promising and innovative intervention, the study did not clarify whether the observed benefits were attributable to AS tears, other treatments, or their combination, precluding conclusions about the efficacy of AS tears alone [43].

## 7. Conclusions

Although AS tears are a valuable therapeutic option for DED, there is no consensus protocol to guide their preparation and use. Based on current data, we endorse the use of 20% AS tears for moderate cases and higher concentrations for more advanced disease. Recent evidence also indicates that conventional diluents, along with existing blood processing and storage conditions, are effective in current practice but remain suboptimal.

This perspective utilizes advances in AS tear therapy over the past three years to provide a framework guiding future research aimed at establishing standardized, evidence-based guidelines that can optimize the therapeutic outcomes. Future randomized controlled trials should directly compare the efficacy of AS tear formulations at different concentrations. Alternative diluents, including levofloxacin-containing eye drops, SH-containing eye drops, and CsA-containing ultra-nano emulsions, may enhance the therapeutic potential of AS tears through both intrinsic properties and synergistic mechanisms, although comparative studies with conventional diluents are important to validate these preliminary findings. Standardization of blood processing and storage conditions should build on existing, well-established protocols. Finally, we acknowledge the novelty and potential of emerging applications, including the combination of AS tears and ERT in perimenopausal women, AS tears with topical insulin eye drops, and the use of AS tears for perioperative prophylaxis in patients with GVHD undergoing cataract surgery, and we suggest that further research is needed to evaluate efficacy prior to integration into routine clinical practice.

## Figures and Tables

**Table 1:** Overview of clinical studies since January 2022 evaluating autologous serum tears in DED.

Studies	Type of Study	Number of Patients	Dry Eye Disease (DED) Severity	Autologous Serum (AS) Tears (%)	Comparator Relevant to the Aim of This Perspective	Methods	Statistically Significant Outcomes
Jiang et al. [21] (2025)	Retrospective cohort study	111	Not reported	20% or 50%	Baseline	Subjective symptoms, Schirmer’s test (ST), tear break-up time (TBUT), ocular surface staining (OSS), conjunctival hyperemia, conjunctival impression cytology (CIC)	Improvement in subjective symptoms, ST, TBUT, OSS, conjunctival hyperemia, CIC
Li et al. [22] (2025)	Randomized controlled trial	200	Severe	20%	Baseline	ST, TBUT, OSS, ocular surface inflammatory factors, tear components	Improvement in ST, TBUT, OSS, ocular surface inflammatory factors, tear components
Wang et al. [23] (2025)	Randomized controlled trial	150	Mild-to-moderate	20%	Baseline	Subjective symptoms, ST, TBUT, OSS, corneal sensation, tear inflammatory factors, adverse events	Improvement in subjective symptoms, ST, TBUT, OSS, corneal sensation, tear inflammatory factors
Bachtalia et al. [6] (2025)	Randomized controlled contralateral trial	10	Severe	50%	Baseline	ST, TBUT, OSS, in vivo confocal microscopy (IVCM), visual acuity (VA), adverse events	Improvement in ST, TBUT, OSS, IVCM parameters, VA
Jongkhajornpong et al. [24,25] (2024)	Randomized non-inferiority trial	96	Moderate-to-severe	100%	Baseline	Subjective symptoms, ST, TBUT, OSS, Meibomian gland parameters, adverse events	Improvement in subjective symptoms, OSS
Özlük et al. [26] (2024)	Randomized comparative trial	42	Severe	20%	Baseline	Subjective symptoms, ST, TBUT, OSS, VA	Improvement in subjective symptoms, ST, TBUT, OSS
Shaheen et al. [27] (2024)	Prospective comparative study	40	Not reported	50%	Baseline	TBUT, OSS, VA	Improvements in TBUT, OSS, VA
Kang et al. [28] (2023)	Randomized comparative trial	30	Not reported	20%	Baseline	Subjective symptoms, ST, TBUT, OSS, CIC, VA, adverse events	Improvement in TBUT, OSS
Zheng et al. [29] (2023)	Randomized controlled trial	216	Moderate-to-severe	20%	Baseline	Subjective symptoms, ST, TBUT, OSS, CIC, adverse events	Improvement in ST, TBUT, OSS, CIC parameters
Wróbel-Dudzińska et al. [18] (2023)	Prospective comparative study	22	Severe	100%	Baseline	Subjective symptoms, ST, TBUT, OSS, conjunctival hyperemia, Meibomian gland parameters, VA, adverse events	Improvements in subjective symptoms, ST, TBUT, OSS, conjunctival hyperemia, VA
Neubauer et al. [30] (2023)	Prospective study	23	Severe	20%	20% AS tears with different diluent	Subjective symptoms, ST, TBUT, OSS, tear meniscus height (TMH)	Outcomes not interpretable relative to baseline
Kumari et al. [31] (2023)	Randomized comparative trial	80	Moderate	20%	Baseline	Subjective symptoms, ST, TBUT, OSS	Improvements in subjective symptoms, ST, TBUT, OS
			Moderate	50%	Baseline	Subjective symptoms, ST, TBUT, OSS	Improvements in subjective symptoms, ST, TBUT, OS
			Moderate	50%	20%	Subjective symptoms, ST, TBUT, OSS	Improvements in ST, OS
			Severe	20%	Baseline	Subjective symptoms, ST, TBUT, OSS	Improvements in subjective symptoms
			Severe	50%	Baseline	Subjective symptoms, ST, TBUT, OSS	Improvements in subjective symptoms, ST, TBUT, OS
			Severe	50%	20%	Subjective symptoms, ST, TBUT, OSS	Improvements in ST, TBUT, OS
Berhuni et al. [32] (2022)	Randomized controlled trial	65	Not reported	20%	Baseline	Subjective symptoms, ST, TBUT	Improvements in subjective symptoms, ST, TBUT
Metheetrairut et al. [33] (2022)	Randomized comparative trial	10	Not reported	20%	Baseline	Subjective symptoms, ST, TBUT, OSS, adverse events	Improvements in subjective symptoms
Calvo-de-Mora et al. [34] (2022)	Randomized comparative trial	63	Severe	20%	Baseline	Subjective symptoms, ST, TBUT, OSS, adverse events	Improvements in subjective symptoms, ST, TBUT, OS
Elsayed [35] (2022)	Prospective comparative study	40	Not reported	20%	Baseline	ST, TBUT, OSS, VA, adverse events	Improvements in ST, TBUT, OSS, VA

Abbreviations: AS, Autologous Serum; CIC, Conjunctival Impression Cytology; DED, Dry Eye Disease; IVCM, In Vivo Confocal Microscopy; OSS, Ocular Surface Staining; ST, Schirmer’s Test; TBUT, Tear Break-Up Time; TMH, Tear Meniscus Height; VA, Visual Acuity.

**Table 2:** Overview of clinical and in vitro studies since 2022 evaluating alternative diluents for autologous serum tears in DED.

Studies	Type of Study	Number of Patients	DED Severity	Diluent	Comparator Relevant to the Aim of This Perspective	Methods	Statistically Significant Outcomes
Li et al. [22] (2025)	Randomized controlled trial	200	Severe	Levofloxacin containing-eye drops	Baseline	ST, TBUT, OSS, ocular surface inflammatory factors, tear components	Improvement in ST, TBUT, OSS, ocular surface inflammatory factors, tear components
Wang et al. [23] (2025)	Randomized controlled trial	150	Mild-to-moderate	Levofloxacin containing-eye drops	Baseline	Subjective symptoms, ST, TBUT, OSS, corneal sensation, tear inflammatory factors, adverse events	Improvement in subjective symptoms, ST, TBUT, OSS, corneal sensation, tear inflammatory factors
Bachtalia et al. [6] (2025)	Randomized controlled trial	10	Severe	Artificial tears	Baseline	ST, TBUT, OSS, IVCM, VA, adverse events	Improvement in ST, TBUT, OSS, IVCM parameters, VA
					Artificial tears monotherapy	ST, TBUT, OSS, IVCM, VA, adverse events	Improvement in ST, TBUT, OSS, IVCM parameters, VA
Sharma et al. [37] (2024)	In vitro study—Human primary limbal epithelial cells (LEpiC)	Not applicable	Not applicable	0.1% sodium hyaluronate (SH)-containing eye drops	SH	Anti-inflammatory effects, cell viability	Comparable effects on inflammation reduction, cell viability enhancement
				0.05% cyclosporine A (CsA)-containing ultra-nano emulsions	CsA	Anti-inflammatory effects, cell viability	Comparable effects on inflammation reduction cell viability enhancement
Kang et al. [28] (2023)	Randomized comparative trial	30	Not reported	0.1% SH-containing eye drops	Baseline	Subjective symptoms, ST, TBUT, OSS, CIC, VA, adverse events	Improvement in TBUT, OSS
Neubauer et al. [30] (2023)	Prospective study	23	Severe	Methylcellulose	AS tears with 0.9% saline solution	Subjective symptoms, ST, TBUT, OSS, TMH	Comparable improvement in subjective symptoms, TBUT, ST, OSS, TMH. Fewer instillations required. Less cost-effective formulation.

Abbreviations: AS, Autologous Serum; CIC, Conjunctival Impression Cytology; CsA, Cyclosporine A; DED, Dry Eye Disease; IVCM, In Vivo Confocal Microscopy; OSS, Ocular Surface Staining; SH, Sodium Hyaluronate; ST, Schirmer’s Test; TBUT, Tear Break-Up Time; TMH, Tear Meniscus Height; VA, Visual Acuity.

## Data Availability

No new data were created or analyzed in this study. Data sharing is not applicable to this article.

## Abbreviations

The following abbreviations are used in this manuscript:

AS	Autologous Serum
BSS	Balanced Salt Solution
CIC	Conjunctival Impression Cytology
CsA	Cyclosporine A
DED	Dry Eye Disease
EGF	Epidermal Growth Factor
ERT	Estrogen Replacement Therapy
GVHD	Graft-Versus-Host Disease
IGF-1	Insulin-Like Growth Factor 1
IL-1β	Interleukin 1 β
IL-6	Interleukin 6
IVCM	In Vivo Confocal Microscopy
OSS	Ocular Surface Staining
PDGF-AB	Platelet-Derived Growth Factor AB
PGE2	Prostaglandin E2
rpm	revolutions per minute
SH	Sodium Hyaluronate
sIgA	Secreted Immunoglobulin A
ST	Schirmer’s Test
TBUT	Tear Break-Up Time
TFOS DEWS	Tear Film & Ocular Surface Society’s Dry Eye Workshop
TGF-β	Transforming Growth Factor β
TMH	Tear Meniscus Height
TNF-α	Tumor Necrosis Factor α
VA	Visual Acuity
VEGF	Vascular Endothelial Growth Factor
